# Genetic architecture and genomic selection of female reproduction traits in rainbow trout

**DOI:** 10.1186/s12864-020-06955-7

**Published:** 2020-08-14

**Authors:** J. D’Ambrosio, R. Morvezen, S. Brard-Fudulea, A. Bestin, A. Acin Perez, D. Guéméné, C. Poncet, P. Haffray, M. Dupont-Nivet, F. Phocas

**Affiliations:** 1 Université Paris-Saclay, INRAE, AgroParisTech, GABI, 78350 Jouy-en-Josas, France; 2 SYSAAF, Station INRAE-LPGP, Campus de Beaulieu, 35042 Rennes cedex, France; 3 SYSAAF, Section Avicole, Centre INRAE Val de Loire, 37380 Nouzilly, France; 4 Viviers de Sarrance, Pisciculture Labedan, 64490 Sarrance, France; 5 Université Clermont-Auvergne, INRAE, GDEC, 63039 Clermont-Ferrand, France

**Keywords:** Fish, Heritability, GWAS, QTL, Body weight, Spawning date, Spawn weight, Egg weight, Egg size, Fecundity

## Abstract

**Background:**

Rainbow trout is a significant fish farming species under temperate climates. Female reproduction traits play an important role in the economy of breeding companies with the sale of fertilized eggs. The objectives of this study are threefold: to estimate the genetic parameters of female reproduction traits, to determine the genetic architecture of these traits by the identification of quantitative trait loci (QTL), and to assess the expected efficiency of a pedigree-based selection (BLUP) or genomic selection for these traits.

**Results:**

A pedigreed population of 1343 trout were genotyped for 57,000 SNP markers and phenotyped for seven traits at 2 years of age: spawning date, female body weight before and after spawning, the spawn weight and the egg number of the spawn, the egg average weight and average diameter. Genetic parameters were estimated in multi-trait linear animal models. Heritability estimates were moderate, varying from 0.27 to 0.44. The female body weight was not genetically correlated to any of the reproduction traits. Spawn weight showed strong and favourable genetic correlation with the number of eggs in the spawn and individual egg size traits, but the egg number was uncorrelated to the egg size traits. The genome-wide association studies showed that all traits were very polygenic since less than 10% of the genetic variance was explained by the cumulative effects of the QTLs: for any trait, only 2 to 4 QTLs were detected that explained in-between 1 and 3% of the genetic variance. Genomic selection based on a reference population of only one thousand individuals related to candidates would improve the efficiency of BLUP selection from 16 to 37% depending on traits.

**Conclusions:**

Our genetic parameter estimates made unlikely the hypothesis that selection for growth could induce any indirect improvement for female reproduction traits. It is thus important to consider direct selection for spawn weight for improving egg production traits in rainbow trout breeding programs. Due to the low proportion of genetic variance explained by the few QTLs detected for each reproduction traits, marker assisted selection cannot be effective. However genomic selection would allow significant gains of accuracy compared to pedigree-based selection.

## Background

Rainbow trout (*Oncorhynchus mykiss*) is a worldwide cultured salmonid species with numerous breeding programs implemented over the last 50 years in closed populations. Breeding goals, i.e., the number, the nature and the importance given to the selected traits, are an essential feature of a breeding program because this determines the direction and extent of genetic trends in the population under selective breeding. The traits of interest can be recorded either directly on candidates for selection, as in the case of growth and morphology traits or on their sibs when the trait measurement requires a fish to be killed, such as assessment of disease resistance or processing yield. Until the 2000s, commercial lines have been mainly selected for growth traits by mass selection and external morphology assisted by ultrasound to improve gutted yield [1]. Since then, sib-based selection to improve more efficiently gutted and fillet yields and disease resistance traits have been more and more emphasized [2, 3].

The egg productivity of rainbow trout has so far received limited attention in commercial selective breeding because its high relative fecundity (1500 to 2000 eggs / kg of body weight) was not considered as a limiting factor [4]. However these traits play an important role in the economics of a breeding company because of the sale of eyed eggs. In addition, the production of caviar from trout eggs is now a developing market, so an increase in the egg number per ton of fish produced and the limitation of egg size would allow an increase in this production.

Reproduction is the basis of all animal production systems. Maintaining or improving reproductive efficiency is essential to the productivity of animal farming. A high reproductive capacity is also one of the main levers for the genetic improvement of the species. It is therefore important to estimate the performance and the genetic trends for reproduction traits, especially since genetic antagonisms are feared with production traits in all species [5].

High selection for production traits in closed and small broodstock populations of rainbow trout have also induced significant levels of inbreeding [6]. Previous works showed significant inbreeding depression effects in female body weight and spawn weight [7] as well as in egg number and spawning age of rainbow trout [8]. Therefore, it is important to evaluate the performance for those female reproduction traits and the possibilities of selecting them in current broodstock.

Early literature reported estimates of genetic parameters for egg production trait in rainbow trout [9–12] or Atlantic salmon [12] based on full-sib analysis of variance of a limited number of families and populations. Only three publications (in rainbow trout: Su et al. [13, 14]; in Coho salmon: Gall and Neira [15]) reported estimates based on REML procedures and animal models that prevent the well-known issue of biased estimation under full-sib analysis of variance.

Since early 1990’s, several breeding programs were developed in France based on combined mass selection on growth and sib selection to improve processing traits and, more recently, disease resistance [1, 16]. The aims of the study were to estimate genetic parameters, to identify quantitative trait loci (QTL) and to assess the efficiency of genomic selection (GS) compared to pedigree-based BLUP selection for female reproduction traits in one of these French rainbow trout selected line. Concerning this last objective, change in selection efficiency was investigated according to two main factors of variation: the size of the reference population and the degree of kinship between reference and candidate populations for selection. Traits under study concerned female body weight, spawning date, fecundity and egg size. As far as we know, it is the first report on the efficiency of (genomic) selection for fish reproduction traits.

## Results

### Genetic parameters

Estimates of genetic parameters are summarized in Table 1. All heritability estimates were in a medium range of values going from 0.27 to 0.44.

**Table 1:** Heritability (on the diagonal), genetic correlations (above the diagonal) and phenotypic correlations (below the diagonal) for female reproduction and weight traits

Trait	SD	FW	PW	SW	EN	EW	ED
SD	0.27 (0.05)	0.12 (0.15)	0.03 (0.15)	0.34 (0.15)	0.22 (0.16)	0.46 (0.15)	0.51 (0.12)
FW	0.04 (0.04)	0.30 (0.06)	0.99 (0.01)	0.12 (0.15)	0.13 (0.16)	−0.01 (0.16)	−0.01 (0.15)
PW	0.04 (0.04)	0.96 (0.01)	0.33 (0.06)	−0.06 (0.15)	−0.02 (0.16)	− 0.10 (0.16)	−0.12 (0.14)
SW	0.05 (0.04)	0.34 (0.03)	0.17 (0.03)	0.32 (0.06)	0.86 (0.05)	0.49 (0.13)	0.59 (0.10)
EN	0.10 (0.04)	0.31 (0.03)	0.17 (0.03)	0.83 (0.01)	0.24 (0.05)	0.01 (0.17)	0.10 (0.15)
EW	0.06 (0.04)	0.09 (0.03)	0.03 (0.03)	0.33 (0.03)	−0.18 (0.03)	0.27 (0.06)	0.99 (0.02)
ED	0.13 (0.04)	0.12 (0.03)	0.05 (0.03)	0.38 (0.03)	0.01 (0.03)	0.70 (0.01)	0.44 (0.07)

Standard errors are given in brackets. When adjusting SW and EN for a constant FW, their genetic correlations with FW dropped to respectively −0.26 (±0.14) and − 0.17 (±0.16), while the phenotypic correlations were close to zero with respective estimates of − 0.05 (±0.04) and 0.06 (±0.04)

*SD* Spawning date*; FW* Female body weight; *SW* Spawn weight; *EN* Egg number; *EW* average egg weight; *ED* Average egg diameter

Our estimates of correlations for the two measures of female body weight with (FW) or without (PW) consideration of the spawning and coelomic liquid weights showed that the two measures were essentially describing the female own weight since we estimated a phenotypic correlation between FW and PW of 0.96 with a genetic correlation close to unity. Regarding correlations between reproduction traits, it should be first mentioned that genetically speaking, EN and SW on one hand, and EW and ED on the other hand corresponded to almost the same traits with genetic correlations (rg) estimated to 0.86 (±0.05) and 0.99 (±0.02), respectively. All those high correlations are partly due to the auto-correlations originating from methods of deriving these traits from common measures of biological components, as shown by the high environmental correlations observed between those pairs of traits (0.82 between EN and SW and 0.55 between EW and ED in our study).

Although EN and SW were highly correlated in our study, those two traits did not behave similarly with ED and EW. On the contrary, ED and EW could be considered as two different measures of a unique biological egg size trait, those two traits being associated in a very similar manner to all the other traits in the analysis. SW was significantly and positively associated with egg size traits (rg in the range 0.5–0.6), while EN was genetically uncorrelated to egg size traits (rg in the range 0.01–0.10) in our study. Both EN and SW traits were genetically weakly correlated to SD (0.22 and 0.34 respectively). Intermediate positive genetic correlations were also estimated between egg size traits and SD.

Regarding the correlations between SD and PW (or FW), we did not observed any significant phenotypic or genetic associations in our study. The female body weights were also not genetically correlated to any of the egg size traits (EW and ED) or egg quantity traits (EN and SW). Considering FW as a covariate in the modelling of EN and SW led to negative genetic correlations between FW and the adjusted EN* and SW* while regressing towards 0 the corresponding phenotypic correlations.

### Genetic architecture

**Fig. 1:**
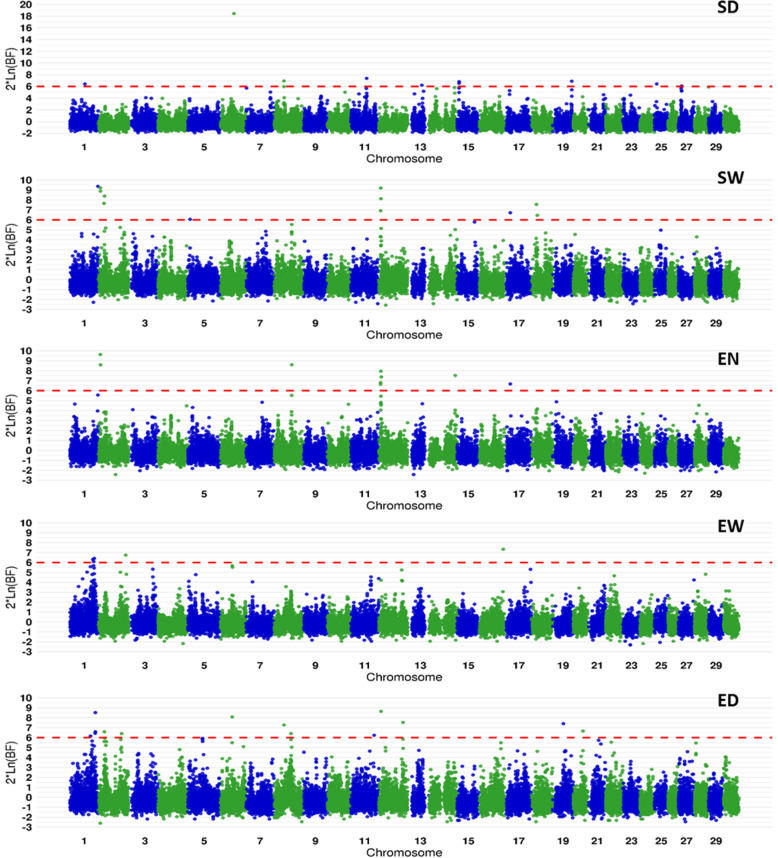
Manhattan plot of QTL detected under Bayesian GWAS for female reproduction traits. The red line corresponds to the threshold logBF > 6.0 for defining evidence for a QTL; SD: spawning date; SW: spawning weight; EN: egg number; EW: egg average weight; ED: egg average diameter

With the only exception of SD, no QTL explained over 3% of the genetic variance (Table 2). Therefore, the genetic architecture of all female reproduction traits appeared to be highly polygenic (Fig. 1 and Supplementary Figure 1, Additional File 1). Only 2 to 4 QTLs were detected that explained at least 1% of the genetic variance for any of the five reproduction traits (Table 2). Ten of the twelve QTLs that were at 1% chromosome-wide significant under GBLUP analysis corresponded to QTLs with strong evidence under the Bayesian approach. In most cases, the same peak SNPs were given by both studies. The consistency of both GWAS was a bit less clear for EW and ED traits. In total, the Bayesian approach allowed the detection of 17 QTLs that explained at least 1% of the genetic variance, but only nine had a very strong evidence (logBF > 8).

**Table 2:** Summary statistics for GWAS for female reproduction traits based on GBLUP and BayesCπ methods

Trait	Peak SNP			GBLUP		BayesCπ		
	Omy	Identifier	Position (bp)	-log(***P***-value)	Confidence interval (Mb)	LogBF	Credibility interval (Mb)	Var^a^ (%)
**SD**	6	Affx-88,920,061	38,611,684	11.5	38.61–38.61	18.4	38.61–38.61	7.2
**SD**	11	Affx-88,936,987	44,670,771			7.4	43.17–45.32	1.9
**SD**	15	Affx-88,942,247	5,640,348			6.8	4.86–5.64	1.2
**SD**	27	Affx-88,907,414	8,854,196			6.1	7.04–8.95	1.7
**SW**	1	Affx-88,918,810	79,053,674			9.4	77.68–79.05	1.3
**SW**	2	Affx-88,951,720	5,801,797	6.3	5.80–6.18	9.2	5.80–6.18	2.9
**SW**	2	Affx-88,927,743	17,660,020	6.1	16.29–17.66	8.4	16.29–17.66	1.9
**SW**	12	Affx-88,950,456	5,349,425	6.9	4.56–6.22	9.2	4.56–6.80	2.5
**EN**	2	Affx-88,951,720	5,801,797	6.5	5.80–6.18	9.6	5.80–6.18	3.2
**EN**	8	Affx-88,953,704	50,018,409	5.9	49.64–50.02	8.6	49.64–50.02	1.4
**EN**	12	Affx-88,950,456	5,349,425	6.2	4.23–6.80	8.0	4.56–6.80	2.7
**EW**	1	Affx-88,932,157	48,339,143	5.4	48.34–48.60			
**EW**	1	Affx-88,950,822	64,632,546	7.0	60.50–64.63	6.3	61.59–64.63	1.2
**EW**	1	Affx-88,915,911	67,153,692	7.2	66.61–68.88			
**EW**	1	Affx-88,957,820	68,664,516			6.4	66.54–68.88	2.0
**ED**	1	Affx-88,941,218	68,345,854	6.0	68.35–68.66			
**ED**	1	Affx-88,942,198	71,813,379	7.1	70.85–71.81	8.5	70.85–71.81	1.9
**ED**	2	Affx-88,930,958	17,234,307			6.6	16.14–18.25	1.5
**ED**	8	Affx-88,908,356	47,852,976			6.4	47.84–49.70	1.6
**ED**	12	Affx-88,930,328	68,199,037			7.5	67.33–68.79	1.1

^a^Var: % of genetic variance explained by all the SNPs included in the QTL credibility interval

*SD* Spawning date; *SW* Spawn weight; *EN* Egg number; *EW* Egg average weight; *ED* Egg average diameter

### Genomic selection

**Fig. 2:**
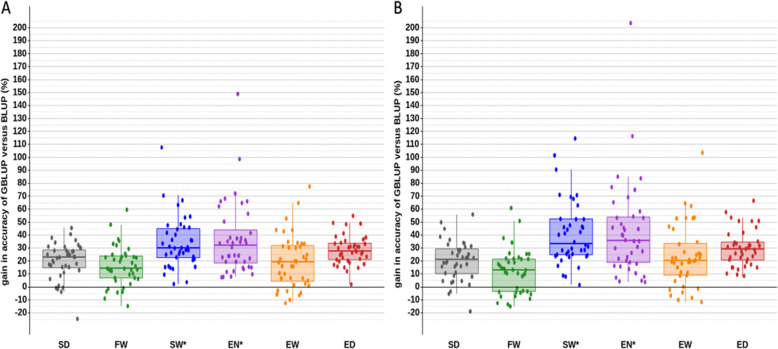
Boxplots of accuracy of GBLUP versus BLUP in 40 simulations with a large training population T+ (A) and a small training population T- (B). SD: spawning date*;* FW: female body weight; SW*: spawning weight (adjusted for FW); EN*: egg number (adjusted for FW); EW: average egg weight; ED: average egg diameter

Efficiency of genomic selection in comparison to pedigree BLUP selection is presented in Fig. 2 and Supplementary Table 1, Additional File 2 for both size scenarios (T- and T+) in terms of training population size (Table 3).

**Table 3:** Number and cohort origin of animals in training and validation sets for cross-validation tests

Scenario	T+	T-	T1	T2
Training set	1077 fish randomly chosen in C1 + C2	672 fish randomly chosen in C1 + C2	726 fish in C1	620 fish in C2
Validation set	269 remaining fish in C1 + C2	same 269 fish as in scenario T+	620 fish in C2	726 fish in C1

Whatever the trait and the scenario, we noticed a slight tendency to overestimate (b < 1) the variation of GEBVs compared to the genetic variance although all inflation coefficients were statistically not different from 1 (see Supplementary Table 1, Additional File 2). The GBLUP accuracy was higher than the BLUP accuracy in most of the 40 validation samples (Fig. 2); less than 10% of the samples failed to respect this rule in average over all the traits. In 60% of the 40 samples, GBLUP was more accurate than BLUP for any of the six traits; loss of accuracy of GBLUP was less than 10% in 80% of the remaining samples.

For T- scenario, the mean accuracy of GEBVs varied from 0.50 for EW to 0.60 for ED whereas the corresponding accuracies of EBVs were 0.41 and 0.47 (see Supplementary Table 1, Additional File 2); when comparing GBLUP versus BLUP, the highest increase in accuracy (+ 35%) was obtained for SW* while the lowest increase (+ 10%) was observed for FW. The same trend was observed for T+ scenario when comparing GBLUP versus BLUP. The mean accuracy of GEBVs varied from 0.55 for EW to 0.66 for ED whereas the corresponding accuracies of EBVs were 0.50 and 0.60 (see Supplementary Table 1, Additional File 2); the highest increase in accuracy (+ 32%) was obtained for SW* while the lowest increase (+ 16%) was observed for FW.

**Fig. 3:**
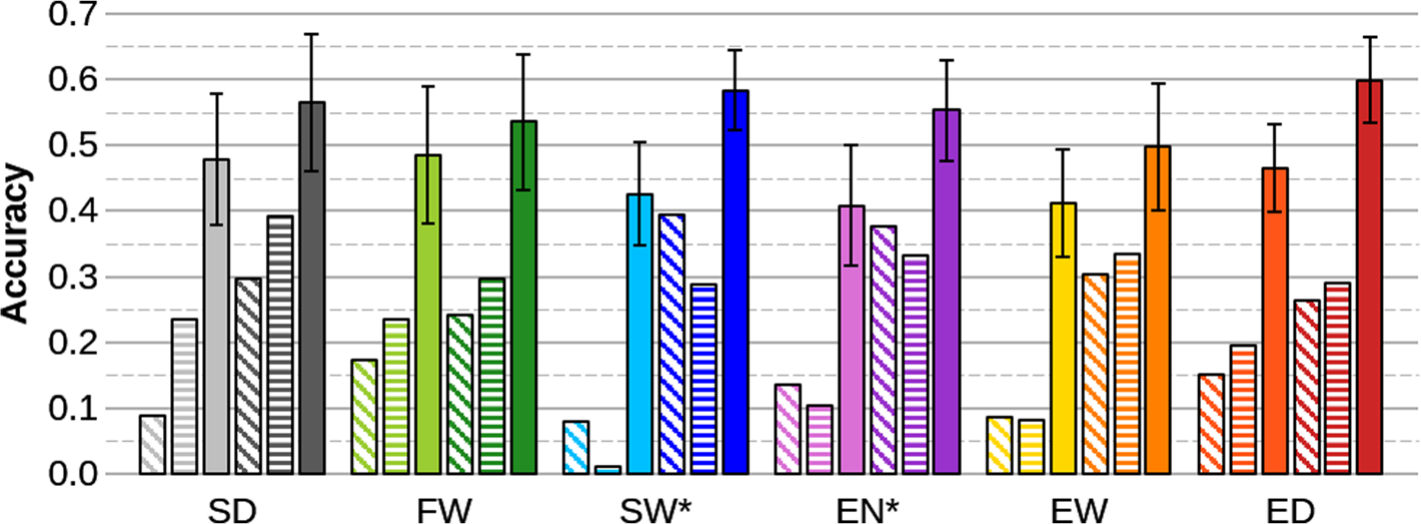
Selection accuracy for BLUP (light colors) or GBLUP (dark colors) considering the training scenarios T1 (diagonal hatching bars), T2 (horizontal hatching bars) and T- (full bars). For scenario T-, the sampling standard deviation over 40 replicates is indicated by a black segment above and below the mean*.* SD: spawning date*;* FW: female body weight; SW*: spawning weight (adjusted for FW); EN*: egg number (adjusted for FW); EW: average egg weight; ED: average egg diameter

For T1 and T2 scenarios, the mean accuracy of GEBVs varied from 0.24 to 0.39 whereas the corresponding accuracies of EBVs were 0.01 and 0.23 (see Supplementary Table 2, Additional File 2). When comparing selection accuracy (Fig. 3) depending on the degree of kinship between the training and the validation populations (T1 or T2 versus T-), we observed that the accuracy was drastically reduced when the training population was less related to the validation population and that the accuracy loss was more severe for BLUP (− 71% in average over the 6 traits) than for GBLUP (− 43%).

## Discussion

### Genetic parameters

The estimated heritability values for FW and PW in the present study are within the range (0.27–0.43) of values reported in recent studies in the same species for female body weight between 13 and 25 months [17–19]. In a study that accounted for random full-sib family effects in an animal model, Su et al. [13] estimated lower heritabilities for PW with values ranging from 0.09 for a control line to 0.26 for a line selected for egg size while the estimate was 0.13 for the line selected for yearling body weight; the full-sib family effect was very significant accounting for 22, 13 and 10% of the phenotypic variance for the three lines, respectively. In our study, no significant full-sib family effect was detected whatever the trait under consideration. A maternal random effect was also tested for all traits, but were never significant. It is likely due to the mixing of eyed-eggs from all the different families and their rearing in common tanks.

Heritability estimated for SD in our study was moderate in comparison to the large values (0.49 to 0.87 with a pooled heritability of 0.65) estimated by Su et al. [13] for their three rainbow trout lines bred derived from the same experimental base population of University of California. However, Gall and Neira [15] estimated a moderate heritability (0.24) for SD in Coho salmon that may be partially explained by the fact all the genetic variation was not accounted for because the spawned females represented a selected group of earliest spawners. The same explanation may also be considered to understand our own moderate estimate of heritability for SD in rainbow trout.

All our heritability estimates for rainbow trout reproductive traits (SD but also PW, SW, EN and EW) were in general close to those of Gall and Neira [15] in Coho salmon, although their heritability for EN was higher (0.42 vs 0.24 in our study) and more consistent with their heritability of SW than in our study. Considering EW as a measure of egg size, Gall and Neira [15] found a similar estimate of heritability as ours. In Su et al. [13], egg size was measured as a volume (in pl), neither corresponding to EW or ED. They found high estimates of heritability for their egg size trait with a pooled heritability of 0.60 across their three rainbow trout lines. Rather than measuring SW, Su et al. [13] measured the egg volume (ml) by allowing the eggs to settle in a volumetric cylinder after water hardening; egg size was determined by dividing 30 by the count of eggs in a 30 ml sample. Egg number was then obtained by dividing egg volume by egg size and its pooled heritability was very high (0.55) compared to ours and in a lesser extent to Gall and Neira [15]‘s one.

Regarding correlations between reproduction traits, the high correlation we estimated between EN and SW was also observed in Coho salmon [15]. However these authors estimated a large negative genetic correlation (− 0.63) between EN and EW for Coho salmon while we did not observed any significant correlations between EN and egg size traits in our study. Our result was only consistent with Su et al. [13]’ one for this particular point. Therefore the question is still pending whether or not selection for egg size may cause or not a decrease in egg number in salmonids or if the negative correlation that we observed is only population specific.

Considering genetic correlations between egg size traits and SD, our estimates were consistent with the estimate (0.51) in Su et al. [13]’ study while Gall and Neira [15] reported a weaker (0.16) genetic correlation in Coho salmon. The positive, but weak, genetic correlations we estimated between egg quantity traits (EN and SW) and SD were also reported by Su et al. [13] and Gall and Neira [15] between EN and SD. However in Coho salmon the genetic correlation estimated between SW and SD was null [15].

Regarding the correlations between SD and female body weights, our results were in contradiction with the significant positive genetic correlation (0.51) reported by Su et al. [13] but very consistent with those for Coho salmon [15]. This may be due to the inclusion of only early spawning females in our study as in Gall and Neira’ one.

Although female body weights did not significantly correlated neither to egg quantity traits (EN, SW) or egg size traits (EW, ED) in our study, significant positive genetic correlations were found for PW with EN (0.47), egg size (0.51) and egg volume (0.67) in US lines of rainbow trout [13]. Gall and Neira [15] reported also in Coho salmon that PW correlated moderately with EN (0.32) or EW (0.37), and more strongly with SW (0.56). Therefore our results are not consistent with the two previous studies in salmonid species regarding genetic associations between female body weight and egg production traits.

Earlier studies indicated that selection for body weight at any age would result in a correlated response for body weight at other ages including age at slaughter and age at spawning for females [20] and that genetic correlations between yearling weight, post-spawning weight of females and egg size, egg number and egg volume were moderate but positive in rainbow trout [14]. These findings led to the assumption that breeding programs designed to increase body size would result in improving egg production traits [14]. However our results do not really validate this assumption since FW and PW do not appear to be genetically correlated - neither positively nor negatively - to any of the reproduction traits (SD, SW, EN, EW or ED). It is therefore important to consider direct selection for improving egg production traits in rainbow trout breeding programs.

### Genetic architecture

The credibility/confidence intervals associated with the QTLs were large in most cases, which precluded the meaningful identification of potential underlying candidate genes that may explain the phenotypic variations observed for female reproduction traits. Nevertheless, based on the functional information given in the human gene database GeneCards® [21, 22] and described phenotypes in mutant mice or worms, we were able to propose five candidate genes for female reproduction traits (3 for SD, 1 for ED and 1 shared by EN and SW). No phenotypes were described for these candidate genes in the zebrafish database ZFIN [23]. As far as we know, our study is the first report for QTLs and candidate genes playing for fecundity and egg size traits in rainbow trout.

Fitness traits such as spawning date and body weight are major factors in the life history of salmonid fishes. Despite the fact that some recent QTLs studies have focused on growth traits in rainbow trout [17, 19] the only significant SNP we detected for female body weight was not in the vicinity of any QTL regions reported for trout body weight. This SNP was not presented in Table 2 because the detected association was considered as a spurious one. There was indeed no evidence for a QTL under GBLUP analysis and only a single SNP was detected on Omy1 with a logBF > 6 in an intergenic region between crocc2 and slco1f1 genes that are not annotated as playing a role in growth function.

In our study, the highest significant (*p*-value < 10^− 6^ at the genome level) SNP was found for SD on Omy6. It corresponds to the only significant QTL for SD under GBLUP analysis and this single SNP explained over 7% of the genetic variance under BayesC approach. The SNP Affx-88,920,061 is located within the importin-11 gene (alias Ran-Binding Protein 11) that plays a receptor role in nuclear protein import. The phenotypes observed in a MGI mouse strain (ID MGI:5617259) with a mutation in this gene may help to understand the effects of a variant allele may have on spawning date in rainbow trout. The double mutant homozygote is a pre-weaning lethal phenotype in mouse and the heterozygote exhibits decreasing levels of iron and glucose levels in the blood. In addition, the heterozygote male had an abnormal eye (lens) morphology. However we may wonder whether the association is a spurious one due to a very high rate of mendelian errors at this SNP position when comparing genotypes from progeny and their parents. Indeed, 104 mendelian errors were observed at this SNP while the medium number of mendelian errors was only 3 for the 4067 SNPs (out of 27,799 SNPs) with at least one mendelian error detected.

While the genetic architecture underlying SD is still largely unknown, a lot of QTLs affecting SD or age at sexual maturation have been detected in salmonid species during the last 20 years [24–30]. Due to the low density of markers and the non standardisation of linkage group names in early studies, it is difficult to report whether our QTLs may have been detected in other studies. Nevertheless, as far as we know, no QTL for SD in salmonid species has been reported in the neighborhood of the highly significant SNP we observed on Omy6. Under Bayesian GWAS, three others QTLs for SD were detected on Omy11, Omy15 and Omy27. Each of them explained between 1.2 and 1.9% of the genetic variance of SD. There is no obvious candidate gene for the large QTL region on Omy11. On the contrary, it is worth mentioning that the peak SNP on Omy15 is positioned within the ARHGEF4 (Rho Guanine Nucleotide Exchange Factor 4) gene that acts as guanine nucleotide exchange factor (GEF) for RHOA, RAC1 and CDC42 GTPases. MGI mutant phenotypes for ARHGEF4 concern in particular immune system and metabolism with decreased hemoglobin content, decreased IgE level, decreased IgG1 level, decreased T cell number, increased mature B cell number, increased circulating alkaline phosphatase level, increased circulating total protein level and decreased circulating triglyceride level. Despite the large credibility interval for the last QTL on Omy27, we may also propose NR2E1 as a convincing candidate gene because the peak SNP is located just behind this estrogen-related receptor gamma-like gene. This gene is an orphan receptor that binds DNA as a monomer to hormone response elements and is in particular involved in the regulation of retinal development and essential for vision. It may be involved in retinoic acid receptor regulation in retinal cells. MGI mutant phenotypes for NR2E1 have abnormal optic nerve and retina morphology, abnormal brain morphology, decreased body size and total body fat amount and decreased female fertility. Interestingly, we can hypothesize that two of the four QTLs detected for SD may be associated to abnormal eye morphology and defects in vision that may render trout less sensitive to the photoperiod stimuli.

Concerning the fecundity traits EN and SW, two QTLs sharing the same SNP peaks on Omy2 (Affx-88,951,720) and Omy12 (Affx-88,950,456) were detected, confirming that the two traits are biologically very close. The QTL on Omy2 explained 3.2 and 3.0% of the genetic variance for EN and SW, respectively. The QTL on Omy12 explained about 2.5–2.7% of the genetic variance for each trait. For EN, a third QTL was detected on Omy8 that explained 1.4% of the genetic variance. No obvious gene candidate could be proposed for this QTL. For SW, a third QTL was detected on Omy2 that explained 1.9% of the genetic variance and a last QTL was detected on Omy1 that explained 1.3% of the genetic variance. These last two QTLs for SW had large credibility intervals (> 1 Mb) and no obvious candidate gene could be proposed. Concerning the common peak SNP for the QTL on Omy2 shared by EN and SW, its location is in-between the MPRD gene (cation-dependent mannose-6-phosphate receptor-like) and the PHC1 gene (polyhomeotic-like protein 1). This PHC1 gene is a homolog of the Drosophila polyhomeotic gene, which is a member of the Polycomb group of genes. It is a component of a Polycomb group (PcG) multiprotein PRC1-like complex, a complex class required to maintain the transcriptionally repressive state of many genes, including Hox genes, throughout development [31]. MGI homozygous mutant phenotypes for the PHC1 gene exhibit perinatal lethality, posterior skeletal transformations and defects in neural crest derived tissues, including ocular abnormalities, cleft palate, parathyroid and thymic hypoplasia and cardiac anomalies. We hypothesize that this gene may have a role in rainbow trout fecundity.

For egg size traits measured by EW and ED respectively, we will focus on the three QTLs on Omy1 showing the highest consistency of results across GWAS (Table 2). Among those QTLs, the same QTL region on Omy1, spanning between 66.539 Mb and 68.881 Mb was detected for the two traits, although the peak SNPs were different across GWAS (67.15 Mb for GBLUP and 68.66 Mb for BayesCπ). No obvious candidate gene could be proposed in this large QTL region that explained 2% of the genetic variance for EW.

Regarding EW, there was a strong evidence for a distinct QTL explaining 1.2% of the genetic variance, in the region spanning from 60.498 Mb and 64.633 Mb on Omy1 with the peak SNP very close to 64.633 Mb in an uncharacterized protein (LOC110527930). No candidate gene could be proposed within this QTL region, but in the close vicinity of this QTL region, let us mention the presence of the prkg2 gene (located between 64.660 and 64.676 Mb on Omy1) whose role is important in oocyte maturation in mammals and zebrafish [32]. This prkg2 gene may be therefore suggested as a functional candidate gene.

Regarding ED, there was evidence for another QTL on Omy1 that explained nearly 2% of the genetic variance in the region spanning between 70.848 Mb and 71.813 Mb. The peak SNP (at 71.813 Mb) is close to the position (71.675–71.699 Mb) of the WAPLA gene (wings apart-like protein homolog) which is a straightforward gene candidate for explaining this last QTL on Omy1. Indeed this gene is a regulator of meiotic chromosome structure and function, playing a role in sister chromatid cohesion, cohesin association with chromatin, DNA double strand break repair and polar body positioning following meiotic divisions during oogenesis [33, 34]. Worm *C. elegans* mutants have an egg-laying defect and reduced brood size with 21% displaying embryonic lethality whilst 28% arrest at the larval stage [34].

### Genomic selection

The implementation of genomic selection in a breeding program for female reproduction traits would allow a gain in accuracy of 16 to 32% (depending on the trait studied) compared to BLUP selection when considering a reference population of about 1100 individuals. For a smaller reference population of 670 individuals, genomic selection will also be more efficient than pedigree-based selection. In the literature, several studies in salmonids [17, 35–38] reported moderate to strong gains in accuracy (+ 11% to 110%) for genomic selection compared to BLUP selection of depending on the genetic architecture of the traits and the size of the reference populations. Increasing by 60% the size of the training population (starting from 670 individuals) in a fish line whose effective population size is estimated around 50 [6], increased the accuracy of GEBVs by 6% to 11% depending on the trait considered. Considering Goddard [39] prediction equation of GS accuracy, we could have expect an increase of accuracy of about 20% in our rainbow trout line when increase our training population from 670 to 1070 animals. Significantly higher gain in accuracy (about + 80%) was described for gestation length (h^2^ = 0.4) in a pig line when increasing by 130% the training population size (starting from 550 sows) in a Large White line with effective population size estimated to be about 100 [40].

We showed a severe loss of accuracy when the degree of kinship between the training and the validation populations was less related and this was more important for BLUP than GBLUP. It is well known that close relationships between animals in the training and validation sets increase the accuracy of genomic predictions compared to the ones derived for an independent validation population [41]. Under scenarios T1 and T2, our estimates of GS accuracy are close to the theoretical estimates derived from Goddard [39]’ formula, which makes sense since one of the basic assumption beyond his formula is that GS accuracy comes only from linkage disequilibrium across the whole population and not from any linkage association and family structure. The fact that the loss of accuracy was less severe for GBLUP than for BLUP had also be shown on poultry [42]. This phenomenon has also been quantified in a salmon population dedicated to genomic selection for sea lice resistance [36]. What is less known is that these strong decreases in accuracy are also associated with strong biased in the predictions (see Supplementary Table 2, Additional File 2). While the variance of GEBVs was not significantly overestimated when the training and validation sets were closely related (see Supplementary Table 1, Additional File 2), it became either strongly inflated or deflated when relationships became more distant. The same observation held for BLUP predictions, even in a larger extent. This may be a strong issue to correctly predict genetic trends or performing optimal multitrait index selection since the magnitude of the inflation varied a lot across traits. Nevertheless, when performing selection within cohort based on a training population including sibs of the candidates, it does not appear to be a problem.

## Conclusions

In our study, all female reproduction and weight traits were moderately heritable with spawn weight showing strong and favorable genetic correlations with number of eggs in the spawn and individual egg size traits (egg average diameter or weight). On the contrary, number of eggs in the spawn was uncorrelated to egg size traits and female body weight (just before or after spawning) was not genetically correlated to any of the reproduction traits. Therefore it is unlikely that selection for growth will induce any indirect improvement for female reproduction traits. It is thus important to consider direct selection for spawn weight for improving egg production traits in rainbow trout breeding programs.

The GWAS results suggested that female reproduction traits are highly polygenic. Only six QTLs over the 19 identified across the five traits studied explained at least 2% of the genetic variance. These results suggest that gene-assisted selection will be useless for improving reproduction traits. However, genomic selection based on a reference population of only one thousand individuals related to candidates may improve the efficiency of BLUP selection from 16 to 37% depending on female reproduction traits. Cross-validation test of genomic prediction highlighted the clear increase in prediction accuracy compared to that of pedigree-based prediction in almost all population samples. The accuracy of GBLUP was the highest when training and validation sets were closely related but the relative advantage over pedigree-based prediction within a population was the largest when relationships were more distant.

## Methods

### Phenotypes

**Fig. 4:**
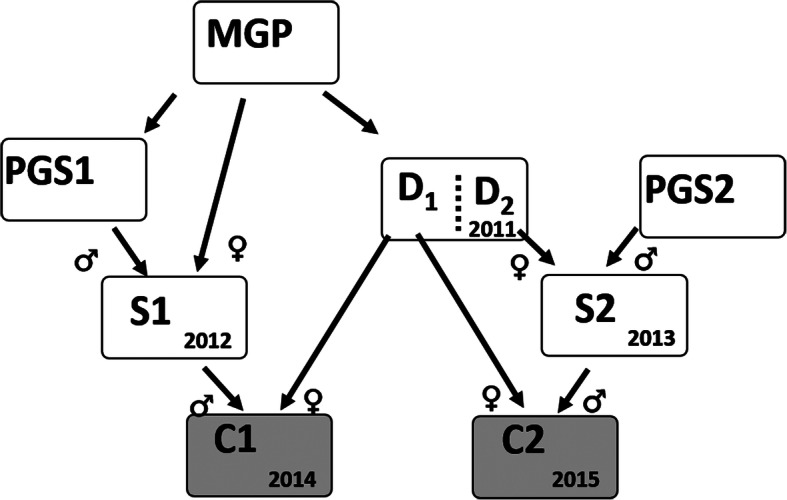
Pedigree structure of cohorts C1 and C2 produced in 2014 and 2015, respectively. MGP: maternal grandparents of C1 and C2; PGS1 (PGS2): paternal grandsires of C1 (C2); D1 (D2) and S1 (S2): dams and sires of C1 (C2)

Phenotypes were collected at 2 years of age in females from two successive cohorts produced in 2014 and 2015, hereafter named C1 and C2, and composing the 9th generation of selection of the trout breeding company “Viviers de Sarrance”. Those cohorts constituted the broodstock of the company and came from two related paternal cohorts S1 and S2 and from different groups of dams D1 and D2 of the same maternal cohort produced in 2011 (Fig. 4). The animals used in the study were reared at the French farm “Viviers de Sarrance” (Pisciculture Labedan, 64,490 Sarrance, France). They were released after the data and egg collection and they reproduced a second time the year after the study.

Raw phenotypes collected were the weight of the ready-to-spawn female (FW), its post-spawning weight (PW), the weight of the total egg mass hereafter called the spawn weight (SW), the length of 50 eggs aligned along a graduated rule, the weight and the number of eggs in a sampling spoon of 2.5 ml, the spawning week number in the calendar year and the presence of overmature eggs in the spawn. All performance records which were more than 4 standard deviations from the mean in absolute value were considered as outliers and discarded from the study. Analyzed traits were the raw phenotypes FW, PW and SW and four derived phenotypes: egg average diameter (ED), egg average weight (EW), egg number of the spawn (EN) and spawning date (SD). ED was calculated as the length of 50 aligned eggs divided by 50. EW was derived as the ratio of the weight of eggs to the number of eggs in the sample of 2.5 ml. EN was calculated as the ratio of SW to EW. SD corresponded to the rank of the week number within the spawning period, with discrete values ranging from 1 (for the first week) to 5 (for the 5th and after weeks) within cohort. In total, the phenotypes of 1517 fish were considered in the study (Table 4).

**Table 4:** Summary statistics of 2-year female reproduction and weight traits in the rainbow trout broodstock

Trait	Number	Mean	SD	Min	Max
Spawning date (SD, week rank)	1517	2.52	1.54	1	5
Ready-to-spawn female body weight (FW, g)	1516	2067	448	1010	3580
Post-spawning body weight (PW, g)	1515	1845	408	840	3116
Spawn weight (SW, g)	1517	187	71	12	398
Egg number (EN, #)	1507	4710	1743	577	10,434
Average egg weight (EW, mg)	1501	39.9	6.5	20.0	68.1
Average egg diameter (ED, mm)	1504	4.03	0.19	3.32	4.72

*SD* Standard deviation

### Genotypes

Among the 1517 phenotyped fish, 1346 fish (726 and 620 individuals from C1 and C2 cohorts, respectively) were genotyped for 57,501 SNPs (Single Nucleotide Polymorphism markers) with the Axiom™ Trout Genotyping array [43] at the INRAE genotyping Platform Gentyane. Most of the fish have their parents also genotyped for the 57,501 SNPs. Only 45 phenotyped fish had ungenotyped dams and 2 other phenotyped fish had ungenotyped sires. Those fish were retained for the genetic analysis considering either unknown dam or unknown sire in their pedigree. The remaining 1301 fish were produced by 83 dams and 71 sires within 7 factorial plans of 12 dams mated to 10 or 11 sires. The genomic relationships among fish were in average 0.05 between cohorts C1 and C2, 0.08 within cohort C1 and 0.07 within cohort C2. The highest genomic relationship values were 0.30 across cohorts, 0.69 and 0.66 within cohort C1 and C2, respectively.

Quality controls of genotyped SNPs were performed as described in D’Ambrosio et al. [6] in particular to remove SNPs with probe polymorphism and multiple locations on the genome assembly (accession number: GCF_002163495.1). Only the 29,799 SNPs with a call rate higher than 0.97, a test of deviation from Hardy-Weinberg equilibrium with a *p*-value > 0.0001 and a minor allele frequency higher than 0.05 were retained for the analysis. All missing genotypes for the 29,799 SNPs were imputed using the FImpute software [44].

### Pedigree-based BLUP-animal model and estimation of genetic parameters

Mixed linear BLUP - animal models were used to get the estimated breeding values (EBV) and to estimate genetic parameters based on pedigree information.

For any trait i among the six reproduction traits considered in the study, the following statistical model was considered to describe the vector of performance **y**_**i**_ of the 1346 fish:

1 $$ {\mathbf{y}}_{\mathrm{i}}={\mathbf{X}}_{\mathrm{i}}{\boldsymbol{\upbeta}}_{\mathrm{i}}+{\mathbf{Z}}_{\mathrm{i}}{\mathbf{u}}_{\mathrm{i}}+{\mathbf{e}}_{\mathrm{i}} $$

where **β**_**i**_, **u**_**i**_ and **e**_**i**_ are the vectors of fixed environmental effects, genetic additive effects and residual effects explaining the performance of all phenotyped animals, respectively. **X and Z** are the incidence matrices for **β**_**i**_ and **u**_**i**_, respectively.

For all the traits, the cohort group (C1 or C2) was considered as the main fixed effect. For the traits FW, SW, EN and ED, an additional fixed effect due to the presence of overmature eggs was significant and therefore considered in the models. The week of spawning had also a significant effect on the traits FW, PW, SW, EN, EW and ED and was considered as a covariate factor nested within cohort.

Because the targeted breeding goal was to increase egg production at a constant weight of female, additional genetic and genomic analyses of SW and EN were performed considering those two traits adjusted (SW* and EN*) for female weight (considered as a covariate factor within cohort).

Tracing back over 8 generations the pedigree of the 1346 phenotyped fish, the vector **u**_**i**_ corresponded to the breeding values of 15,265 individuals related through the pedigree relationship matrix **A**.

To estimate all variance components by Average Information Restricted Maximum Likelihood algorithm [45], eight multi-trait models were run considering four traits in joint analyses. With the female body weight (BW) representing FW in a first set of 4 multi-trait analyses and PW in a second set of 4 multi-trait analyses, the following combinations of traits were considered in the joint analyses: [BW, SD, EW, SW], [BW, SD, EW, EN], [BW, SD, ED, EN] and [BW, SD, ED, SW]. For a given BW, results for any genetic or phenotypic parameter were the averages of 4 estimates, except for the correlations between FW and PW, SW and EN, EW and ED that were estimated under unique bivariate models due to convergence issues related to the very high correlations between those pairs of traits.

The EBV were estimated using BLUPf90 package and the variance components using AIREMLf90 program [46].

### Genomic BLUP model and QTL detection

Genomic BLUP (GBLUP) is an analogous approach to BLUP considering a genomic related matrix **G** [47] in place of a pedigree matrix **A.** The performance modeling is the same as for the BLUP model described in eq. (1), but only the performance of the genotyped animals can be integrated into a conventional GBLUP analysis:

2 $$ {\mathbf{y}}_{\mathrm{i}}={\mathbf{X}}_{\mathrm{i}}{\boldsymbol{\upbeta}}_{\mathrm{i}}+{\mathbf{Z}}_{\mathrm{i}}{\mathbf{g}}_{\mathrm{i}}+{\mathbf{e}}_{\mathrm{i}} $$

with the vector **g**_**i**_ corresponding to the breeding values of 1346 phenotyped and genotyped individuals related through the genomic relationship matrix **G**.

The **G** matrix and the genomic estimated breeding values (GEBV) were estimated using BLUPf90 package [48].

Genome-wide association studies (GWAS) were performed considering the GBLUP models to identify QTL. The postGSF90 module [49] of the BLUPF90 package makes it possible to obtain the estimated effects of the SNPs (**â**_**i**_) from the genomic breeding values ${\hat{\boldsymbol{g}}}_i$ predicted for the genotyped animals according to the equation:

3 $$ {\hat{\boldsymbol{a}}}_i=\boldsymbol{d}\ {\boldsymbol{Z}}^{\prime }{\left[\boldsymbol{Z}\ \boldsymbol{d}\ \boldsymbol{Z}\prime \right]}^{-1}\ \hat{{\boldsymbol{g}}_i} $$

where **d** is the vector of weights associated with the SNP effects.

A region of the genome was considered to be a QTL when the -log_10_(*p*-value) for a SNP of this region was equal or greater than 5.0 (which corresponds to a chromosome-wide significance threshold of 1% derived as -log_10_(0.01/(n/30)) after Bonferroni correction with *n* = 29,799 the total number of SNPs included in the analysis). The peak SNP was considered as very significant when its -log_10_(p-value) was at least equal to -log_10_(0.01/n) = 6.5, corresponding to a genome-wide significance threshold of 1% after Bonferroni correction. Considering a drop-off value of 1.5 log unit of p-value [50], the QTL confidence interval was delimited by integrating all the SNPs into a 1 Mb sliding window around the peak SNP as long as the new SNPs exhibited a -log_10_(p-value) greater than the peak value −1.5.

A Bayes Cπ strategy [51] was used to confirm those QTLs and to better estimate the variance explained by those QTLs. Therefore, a general linear mixture model was defined in which a fraction π of the 30 K SNPs was assumed to have a non-zero effect at each cycle of the MCMC algorithm. A total of 100,000 cycles were performed, with a burn-in period of 5000 cycles. Results were saved every 20 cycles. Convergence was assessed by visual inspection of plots of the posterior density of genetic and residual variances and by deriving high correlations (r > 0.99) between GEBVs estimated from different chains of the MCMC algorithm. Assuming for π a beta distribution B(α,β) with α = 300 and β = 29,800, the π value was kept almost constant at 1%, corresponding to approximatively 300 SNPs selected at each iteration among the 29,800 markers. By trial-and-error, this π value was considered as a good compromise in our variable selection algorithm between the high degree of polygeny of the quantitative traits under study and the limited number of individuals (n ~ 1300) in our dataset that led to consider *p* = 300 < n to correctly estimate p SNP effects simultaneously. We used the BESSiE software [52] to perform the Bayesian analyses.

The degree of association between each SNP and phenotypes was assessed with the Bayes Factor (BF) that involves π and P_*i*_, the probability of the ith SNP to have a non-zero effect: $\mathrm{BF}=\frac{{}^{{\mathrm{P}}_{\mathrm{i}}}/\left(1-{\mathrm{P}}_{\mathrm{i}}\right)}{{}^{\uppi}/\left(1-\uppi \right)}$

As proposed by Kass and Raftery [53], the logBF was computed as twice the natural logarithm of the BF and the threshold logBF ≥6 was used for defining evidence for a QTL. As proposed by Michenet et al. [54], a credibility interval was built around the peak SNP integrating to the QTL region the SNPs with logBF ≥3 that were located close to the peak SNP using a sliding window of 1 Mb on both sides of the peak SNP. Following Michenet et al. [54], we considered that a peak SNP with 6 ≤ logBF < 8 corresponded only to a putative QTL unless the QTL region explained at least 1% of the genetic variance; in that case, the QTL was considered as having a strong effect on the trait of interest.

Candidate genes located within the confidence or credibility intervals estimated using either GBLUP or BCπ analysis were listed from the NCBI *Oncorhynchus mykiss* Annotation Release 100 (GCF_002163495.1).

### Criteria of validation of selection efficiency

To assess the BLUP or GBLUP selection efficiency, cross-validation tests were performed for different scenarios varying the training population size and its relatedness to the validation population. Efficiency was assessed by the accuracy of selection and the inflation coefficient of EBV as a measure of selection bias. The accuracy of selection was derived as the correlation between (G) EBVs of individuals in the validation set and their corrected phenotypes (adjusted for the covariate and fixed effects) divided by the square root of the trait heritability [55]. The inflation coefficient was derived as the regression coefficient of the corrected phenotypes on the (G)EBVs. In the absence of selection bias, this coefficient is expected to be equal to 1; the coefficient value is below 1 in case of EBV over-dispersion (inflation) and the value is above 1 in case of EBV under-dispersion.

To test the training size effect, 40 replicates of Monte-Carlo ‘leave-one-group-out’ cross-validation tests [56] were run. For a given replicate, 269 fish for the validation set, 672 fish for the small training set (T-) and 1077 fish for the large training set (T+) were randomly chosen across the 1346 individuals phenotyped and genotyped. For the T+ and T- scenarios, accuracy of selection and inflation coefficient were derived as the mean over the 40 replicates of the correlation and regression coefficients previously described between (G) EBV and corrected phenotypes for the validation population.

To test the relationship effect between the training and validation populations, the C1 and C2 fish were alternatively used as the training and validation sets, the scenarios T1 and T2 corresponding to C1 and C2 fish in the training sets, respectively (Table 3).

## Supplementary information

**Additional file 1 Supplementary Figure 1**. Manhattan plot of QTL detected under GBLUP-based GWAS for female reproduction traits. The red line corresponds to the genome-wide significance threshold at 1% after Bonferroni correction and the blue line corresponds to the chromosome-wide significance threshold at 1% after Bonferroni correction. SD: spawning date; SW: spawn weight; EN: egg number; EW: average egg weight; ED: average egg diameter

**Additional file 2 Supplementary Table 1**. Mean and standard deviation over 40 replicates (in brackets) of selection accuracy (r) and inflation coefficient (b) of EBVs and GEBVs for training scenarios T+ and T-.SD: spawning date*;* FW: female body weight; SW*: spawn weight (adjusted for FW); EN*: egg number (adjusted for FW); EW: average egg weight; ED: average egg diameter. **Supplementary Table 2**. Selection accuracy (r) and inflation coefficient (b) of EBVs and GEBVs for training scenarios T1 and T2. SD: spawning date*;* FW: female body weight; SW*: spawn weight (adjusted for FW); EN*: egg number (adjusted for FW); EW: average egg weight; ED: average egg diameter.

## Data Availability

The data that support the findings of this study are available from the breeding company « Viviers de Sarrance » but restrictions apply to the availability of these data, which were used under license for the current study, and so are not publicly available. The data can be made available for reproduction of the results from Florence Phocas (florence.phocas@inrae.fr) and Ana Acin-Perez (ana@sarrance.com) on request via a material transfer agreement and with permission of the breeding company « Viviers de Sarrance ». The accession numbers for the rainbow trout reference sequence assembly and the corresponding gene bank assembly were GCF_002163495.1 and GCA_002163495.1 in the National Center for Biotechnology Information database that can be accessed at the weblink: https://www.ncbi.nlm.nih.gov/genome/196?genome_assembly_id=319782 Functional information for genes was extracted from the human gene database GeneCards® at the weblink: https://www.genecards.org/

## Abbreviations

BLUP: Best linear unbiased predictor
EBV: Estimated breeding value
GEBV: Genomic estimated breeding value
GBLUP: Genomic best linear unbiased predictor
GS: Genomic selection
GWAS: Genome-wide association study
MAF: Minor allele frequency
Omy: Chromosome abbreviation for the species *Oncorhynchus mykiss*
QTL: Quantitative trait loci
REML: Restricted maximum likelihood
SNP: Single nucleotide polymorphism
