# Supplementary material for: Genetic architecture and genomic selection of female reproduction traits in rainbow trout
Source: BMC Genomics. 2020 Aug 14;21:558. doi: 10.1186/s12864-020-06955-7 (PMC7430828; doi:10.1186/s12864-020-06955-7)
Supplement: Supplementary file 1 — Additional file 1 Supplementary Figure 1. Manhattan plot of QTL detected under GBLUP-based GWAS for female reproduction traits. The red line corresponds to the genome-wide significance threshold at 1% after Bonferroni correction and the blue line corresponds to the chromosome-wide significance threshold at 1% after Bonferroni correction. SD: spawning date; SW: spawn weight; EN: egg number; EW: average egg weight; ED: average egg diameter [file 12864_2020_6955_MOESM1_ESM.docx]

**Supplementary Figure 1.** Manhattan plot of QTL detected under GBLUP-based GWAS for female reproduction traits.


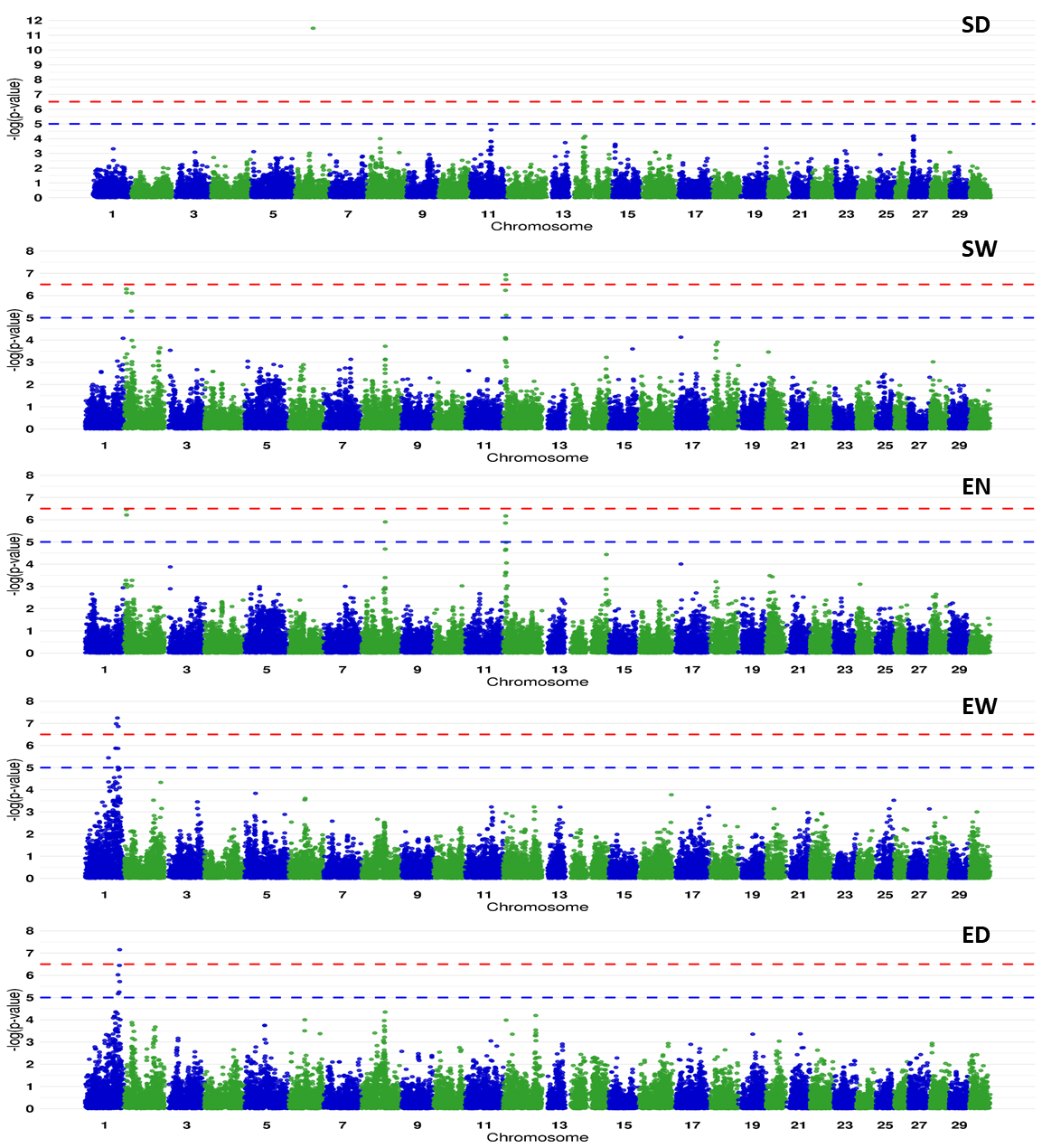
The red line corresponds to the genome-wide significance threshold at 1% after Bonferroni correction and the blue line corresponds to the chromosome-wide significance threshold at 1% after Bonferroni correction.

SD: spawning date; SW: spawn weight; EN: egg number; EW: average egg weight; ED: average egg diameter.
