# Supplementary material for: Genetic architecture and genomic selection of female reproduction traits in rainbow trout
Source: BMC Genomics. 2020 Aug 14;21:558. doi: 10.1186/s12864-020-06955-7 (PMC7430828; doi:10.1186/s12864-020-06955-7)
Supplement: Supplementary file 2 — Additional file 2 Supplementary Table 1. Mean and standard deviation over 40 replicates (in brackets) of selection accuracy (r) and inflation coefficient (b) of EBVs and GEBVs for training scenarios T+ and T-.SD: spawning date; FW: female body weight; SW*: spawn weight (adjusted for FW); EN*: egg number (adjusted for FW); EW: average egg weight; ED: average egg diameter. Supplementary Table 2. Selection accuracy (r) and inflation coefficient (b) of EBVs and GEBVs for training scenarios T1 and T2. SD: spawning date; FW: female body weight; SW*: spawn weight (adjusted for FW); EN*: egg number (adjusted for FW); EW: average egg weight; ED: average egg diameter. [file 12864_2020_6955_MOESM2_ESM.docx]

**Supplementary Table 1.** Mean and standard deviation over 40 replicates (in brackets) of selection accuracy (r) and inflation coefficient (b) of EBVs and GEBVs for scenarios T+ and T- varying the size of the training population.

|  | BLUP | | | | |  | GBLUP | | | | |
| --- | --- | --- | --- | --- | --- | --- | --- | --- | --- | --- | --- |
|  | training population T- | |  | training population T+ | |  | training population T- | |  | training population T+ | |
| Trait | r | b |  | r | b |  | r | b |  | r | b |
| SD | 0.48 (0.12) | 1.01 (0.25) |  | 0.51 (0.10) | 0.97 (0.20) |  | 0.57 (0.12) | 0.92 (0.21) |  | 0.61 (0.10) | 0.92 (0.17) |
| FW | 0.48 (0.11) | 1.05 (0.29) |  | 0.51 (0.10) | 1.01 (0.26) |  | 0.53 (0.10) | 0.96 (0.21) |  | 0.59 (0.10) | 0.95 (0.20) |
| SW* | 0.43 (0.09) | 0.96 (0.21) |  | 0.47 (0.08) | 0.97 (0.19) |  | 0.58 (0.06) | 0.94 (0.12) |  | 0.62 (0.06) | 0.93 (0.12) |
| EN* | 0.41 (0.10) | 0.97 (0.27) |  | 0.45 (0.09) | 0.97 (0.23) |  | 0.55 (0.08) | 0.96 (0.14) |  | 0.59 (0.08) | 0.97 (0.15) |
| EW | 0.41 (0.09) | 0.93 (0.20) |  | 0.46 (0.08) | 0.95 (0.19) |  | 0.50 (0.10) | 0.88 (0.17) |  | 0.55 (0.10) | 0.86 (0.15) |
| ED | 0.47 (0.08) | 0.96 (0.19) |  | 0.51 (0.07) | 0.98 (0.15) |  | 0.60 (0.07) | 0.95 (0.14) |  | 0.66 (0.07) | 0.96 (0.12) |

SD: spawning date; FW: female body weight; SW: spawning weight; EN: egg number; EW: average egg weight; ED: average egg diameter. * when adjusting SW and EN for a constant FW.

**Supplementary Table 2.** Selection accuracy (r) and inflation coefficient (b) of EBVs and GEBVs for training population T1 and T2.

|  | BLUP | | | | |  | GBLUP | | | | |
| --- | --- | --- | --- | --- | --- | --- | --- | --- | --- | --- | --- |
|  | training population T1 | |  | training population T2 | |  | training population T1 | |  | training population T2 | |
| Trait | r | b |  | r | b |  | r | b |  | r | b |
| SD | 0.09 | 0.64 |  | 0.23 | 2.64 |  | 0.30 | 0.58 |  | 0.39 | 1.29 |
| FW | 0.17 | 0.88 |  | 0.23 | 2.26 |  | 0.24 | 0.92 |  | 0.30 | 1.25 |
| SW* | 0.08 | 0.74 |  | 0.01 | 0.09 |  | 0.39 | 2.26 |  | 0.29 | 0.69 |
| EN* | 0.13 | 1.03 |  | 0.1 | 0.86 |  | 0.38 | 0.88 |  | 0.33 | 0.88 |
| EW | 0.09 | 1.06 |  | 0.08 | 0.54 |  | 0.30 | 1.05 |  | 0.33 | 0.72 |
| ED | 0.15 | 1.50 |  | 0.19 | 1.36 |  | 0.26 | 1.25 |  | 0.29 | 0.67 |

SD: spawning date; FW: female body weight; SW: spawning weight; EN: egg number; EW: average egg weight; ED: average egg diameter. * when adjusting SW and EN for a constant FW.
